# Genomic Exploration of the Brain in People Infected with HIV—Recent Progress and the Road Ahead

**DOI:** 10.1007/s11904-023-00675-9

**Published:** 2023-11-10

**Authors:** Amara Plaza-Jennings, Schahram Akbarian

**Affiliations:** 1 Department of Psychiatry, Icahn School of Medicine at Mount Sinai, New York, NY 10029 USA; 2 Nash Department of Neuroscience, Icahn School of Medicine at Mount Sinai, New York, NY 10029 USA; 3 Department of Genetics and Genomic Sciences, Icahn School of Medicine at Mount Sinai, New York, NY 10029 USA

**Keywords:** HAND, HIV-associated Neurocognitive Disorders, Microglia-neuronal Interactions, Neurogenomics of HIV, Postmortem, Human Brain

## Abstract

**Purpose of Review:**

The adult human brain harbors billions of microglia and other myeloid and lymphoid cells highly susceptible to HIV infection and retroviral insertion into the nuclear DNA. HIV infection of the brain is important because the brain is a potentially large reservoir site that may be a barrier to HIV cure strategies and because infection can lead to the development of HIV-associated neurocognitive disorder. To better understand both the central nervous system (CNS) reservoir and how it can cause neurologic dysfunction, novel genomic, epigenomic, transcriptomic, and proteomic approaches need to be employed. Several characteristics of the reservoir are important to learn, including where the virus integrates, whether integrated proviruses are intact or defective, whether integrated proviruses can be reactivated from a latent state to seed ongoing infection, and how this all impacts brain function.

**Recent Findings:**

Here, we discuss similarities and differences of viral integration sites between brain and blood and discuss evidence for and against the hypothesis that in the absence of susceptible T-lymphocytes in the periphery, the virus housing in the infected brain is not able to sustain a systemic infection. Moreover, microglia from HIV + brains across a wide range of disease severity appear to share one type of common alteration, which is defined by downregulated expression, and repressive chromosomal compartmentalization, for microglial genes regulating synaptic connectivity.

**Summary:**

Therefore, viral infection of the brain, including in immunocompetent cases with near-normal levels of CD4 blood lymphocytes, could be associated with an early disruption in microglia-dependent neuronal support functions, contributing to cognitive and neurological deficits in people living with HIV.

## Introduction

A major area of focus in human immunodeficiency virus (HIV) research is the viral reservoir. The reservoir is formed because HIV, as a retrovirus, inserts itself into host DNA. Thus, the life cycle and pathogenicity of HIV are intricately linked to human genome biology and nuclear organization. After cell entry and reverse transcription, a particle containing viral DNA and the integrase enzyme encased in capsid protein is transported into the nucleus via the nuclear pore complex [1]. In the nucleus, HIV hijacks host transcription and splicing factors to guide the virus to chromosomal sites facilitative for transcription for integration [2, 3]. After integration, multiple layers of epigenomic regulation, including DNA methylation and histone modifications at the site of genomic insertion, will determine whether HIV is actively transcribed and leading to productive infection, or alternatively entering a latency stage with dormant provirus transiently or permanently silenced in that infected cell [4]. These cells with quiescent provirus are not detectable by the immune system and may persist for years, leading to rapid viral rebound when antiretroviral therapy is withdrawn [5, 6]. It is because of the viral reservoir that a cure for HIV has remained elusive. The reservoir is also believed to contribute to the development of comorbidities, such as cardiovascular disease, kidney disease, and neurocognitive impairment, in people living with HIV (PLWH) [7, 8].

The dual challenges of the HIV reservoir as a barrier to cure and a cause of HIV-associated comorbid disease are exemplified in the brain. HIV infects cells in the brains of all PLWH, predominately myeloid lineage-derived microglia and perivascular macrophages [9], in addition to subsets of T-lymphocytes and other cells from the lymphoid lineage of the hematopoietic system. The brain provides a potentially large viral reservoir of susceptible cells, with microglia alone comprising an estimated 5–10% of all brain cells [10]. Additionally, 20–50% of PLWH develop HIV-associated neurocognitive disorder (HAND), making HAND a highly prevalent comorbidity of HIV [11–14]. While the reservoir has been studied in detail in the blood using advanced sequencing approaches, the same is not true of the brain.

It will be the goal of this review is to summarize genomic studies conducted on the HIV-infected human brain and to highlight important opportunities for applying novel omics approaches to further our understanding of central nervous system (CNS) infection and disease. Our review focuses on two overarching long-term goals: first, to understand the CNS reservoir, with the expectation that this knowledge will aid in the development of HIV cure strategies. Second, to explore genome-wide adaptations in chromatin structure and gene expression after the brain gets infected, with the expectation that such type of knowledge will provide insight into the cellular mechanisms of cognitive dysfunction and neurological symptoms observed in HIV infection.

## Challenges to Studying HIV CNS Infection

In order to discuss strategies for studying the “omics” of HIV infection in the brain, it is first necessary to understand the unique challenges of studying HIV infection in the brain as compared to blood. First, is the availability of samples. Blood is readily available from living patients and can easily be processed and stored such that viable, whole cells can be studied. Blood can also be collected from the same donors over time, allowing for the study of viral reservoir dynamics [15–17]. Brain tissue, however, is almost exclusively available postmortem. It is typically stored either frozen or in paraffin-embedded slices. Both methods cause the destruction of cells, meaning that intact, whole cells cannot be isolated. Studies must then either use in situ approaches in brain slices, which typically limit the number of genes that can be studied, use bulk tissue approaches, which obscure cell-type-specific signatures, or perform studies using nuclei, which can be isolated intact from frozen postmortem brain. However, even using nuclei, only small portions of the transcriptome and proteome can be studied. Additionally, with brain tissue, it is not possible to have multiple samples over time from the same donor to study the evolution of the virus within the brain of a single individual.

Cerebrospinal fluid (CSF) does allow for repeated longitudinal sampling from the same individuals. However, obtaining CSF is much more difficult and potentially more dangerous than drawing blood. While valuable discoveries have been gleaned from studying CSF in HIV infection, some studies suggest that the CSF does not fully reflect what is occurring in the brain parenchyma during HIV infection [18]. Other model systems, such as cell culture models, Simian immunodeficiency models, and rodent models can also provide insights, but none fully recapitulate the human brain [19–21].

## Understanding the CNS Reservoir

### Current Knowledge

It is clear that HIV integrates into cells in the brain and remains there for many years after infection [22–24]. However, it is not clear whether the CNS reservoir reactivates upon withdrawal of combined antiretroviral therapy (cART) and can seed ongoing infection. This distinction is important because it would mean that HIV in the CNS also needs to be targeted by cure strategies, posing an additional barrier given the challenge of targeting therapeutics to the brain. Perhaps the best evidence we have speaking to this issue comes from a recent study by Kincer and colleagues in which they sequenced viral genomes from the CSF of 11 individuals undergoing treatment interruption [25]. They found that viral sequences present in the CSF after treatment interruption came from clonally amplified T-cell tropic virus. They did not find any evidence of macrophage-tropic viral strains, which would suggest reactivation from microglia. Viral outgrowth assays from humanized mouse models have also shown that T-cells can traffic into the brain and serve as replication-competent viral reservoirs [26].

It is also important in this context to mention anecdotal evidence, collected thus far from three subjects with HIV who each received a bone marrow transplant because of hematologic malignancies [27]. These host patients received donor transplants homozygous for a 32-base pair frameshift mutation in the CCR5 receptor gene (CCR5Δ32), which is known to abrogate cell surface expression thereby conveying natural resistance to CCR5-tropic virus variants [28]. These subjects were closely monitored for multiple years after having received the transplant. Despite being off cART for many years in some cases, to date, no evidence for rebound of HIV-1 viremia or rebound of HIV in CSF has emerged [29, 30]. Case reports essentially declare each of these three patients HIV symptom-free [27]. Put together, this data suggests that replication-competent reservoir in the CNS exists in T-cells which have trafficked into the CNS. It is not clear whether this virus is able to escape from the CSF into the periphery. Thus, at least in the case of the few patients cured of HIV, when there is no pool of susceptible T-cells in the periphery, it would be not unreasonable to hypothesize that the virus is not able to sustain itself.

**Fig. 1:**
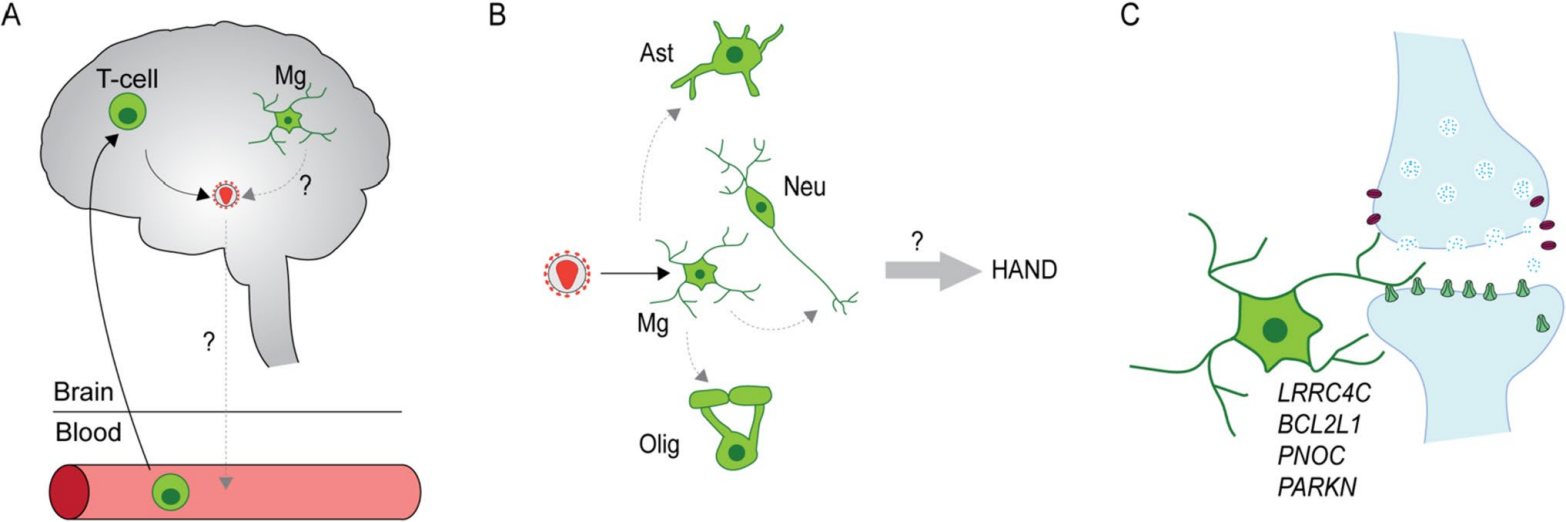
**A** Current understanding of the CNS as a reservoir site. Both microglia and T-cells, which migrated from the periphery are infected in the brain. CNS-resident T-cells have been shown to reactivate and produce virus upon treatment interruption. It is still unknown whether microglia harbor latent replication-competent virus which can reactivate. It is also unknown whether viral reactivation in the CNS can seed ongoing peripheral infection. **B** Current understanding of changes in gene expression contributing to HAND. Microglia are infected, and this can lead to downregulation of microglial genes with a role in neuronal support, including synaptic function. It is unclear how infection impacts astrocytes or oligodendrocytes and how all of these changes together cause HAND. **C** Schematic of microglia interacting with the neuronal synapse. Genes involved in neuronal support that are dysregulated in microglia in HIV infection [23] are indicated

However, the aforementioned people cured from HIV after receiving a genetically engineered bone marrow transplant were exposed to whole body/brain irradiation, and potentially graft vs host disease, and other factors potentially affecting the brain’s viral reservoirs. Therefore, it remains an open question whether other (non-transplant) patients may harbor replication competent virus in microglia or peripheral macrophages capable of seeding continued infection. To date, this has been shown for primary microglia of only a single human brain specimen, and only after treatment of the cultured cells with multiple chromatin modifying drugs designed to broadly activate genomic transcription [31]. However, there have been studies of simian immunodeficiency virus (SIV) showing that HIV exists long-term in a replication-competent state in the myeloid compartment of the non-human primate brain [32]. Of note, studies estimating microglial lifespan and turnover in the human brain, using radiocarbon birth-dating techniques, place the half-life of the “average” microglia at 4.2 years, with 28% renewal rate per year and a subset of cells surviving for more than two decades [33]. Thus, given the available evidence to date, it is reasonable to assume that infected subjects who are cured after receiving the DCCR5 bone marrow transplant and thus have not taken antiretrovirals for many months and years, still harbor replication-competent HIV in a subset of microglial cells that date back to the time period preceding the transplant. We would like to conclude then that, just like discussed previously for the viral rebound in cerebrospinal fluid (CSF) after interruption of antiretroviral treatment [25], any viral reservoirs residing in brain microglia cannot initiate reseeding and spread of infection in the absence of a susceptible or HIV + lymphoid compartment, including CD4 lymphocytes (Fig. 1A).

### Future Work

Future studies are needed to understand whether viral reactivation in the CNS, either from T-cells or microglia and macrophages, can seed peripheral infection and whether reactivation from microglia occurs. As studies of treatment interruption are becoming more common, we suggest that additional studies of CSF and blood are needed. This initial study by Kincer and colleagues [25] revealed very valuable information but was from a small number of participants. Studies of a larger number of individuals are needed to show with more certainty that reactivated virus in the CSF come only from T-cells and not microglia.

The gold standard for the identification of inducible, replication-competent HIV is the quantitative viral outgrowth assay (qVOA) [34]. This methodology requires live cells and thus has not been applied to studying HIV infection in the brain, with one notable exception [31]. However, it is possible to isolate intact microglia from a fresh brain at the time of autopsy [35]. To further study whether replication-competent virus exists in microglia, it will be necessary to perform qVOAs from primary human microglia, especially those from the brains of individuals who have been on long-term cART. Using a similar methodology, it may also be possible to isolate CNS-resident T-cells to study viral reactivation.

Other methods besides the qVOA have been used to suggest the presence of replication-competent virus, such as near full-length proviral sequencing [36, 37]. This can also be paired with RNA-sequencing from the same cell to give information about the transcriptional status of the provirus [17]. While these studies have been done in intact T-cells, there is no reason to think that they could not also be performed in postmortem brain nuclei. However, one challenge regardless of which approach is used is the relative scarcity of infected cells in the brain [23]. A method to enrich for infected cells could greatly enhance the success of these approaches. One approach is the use of fluorescence in situ hybridization paired with flow cytometry (flow-FISH), which has been used to identify cells infected with HIV using RNA FISH [38]. This could also be done using DNA rather than RNA FISH and could be performed in nuclei as well to enrich for infected cells in the brain.

## Viral Integration Site Mapping in the Human Brain—Knowledge State 2023. What are Some of the Tasks Ahead?

### Current Knowledge

In 2022, two studies explored, for the first time, genomic integration in the human (postmortem) brain. The study by Cochrane and colleagues screened human brain tissue from 30 infected donors (and 6 negative controls) with the goal to get some measure for the existence of intact provirus [24]. Towards that end, they harnessed a multiplex PCR strategy with primers directed “inward” within a selected 7564 base pair window internal to the 5′ and 3′ long-term repeat (LTR) flanking sequences of the virus, designed to simultaneously detecting 5′ and 3′ viral sequences in single lipid droplets. This study estimates that in the brain, approximately 90% of proviral DNA sequences are derived from integrants that are defective and hence not replication-competent. However, according to the authors’ estimates, this still leaves an infected person with 5–13 intact viral copies/10^6^ brain cells [24]. Importantly, these numbers were of similar magnitude in brains from donors with efficient antiretroviral therapy-mediated suppression, compared to viremic and hence not fully suppressed cases [24, 39]. Given that the adult human brain harbors 100 billion glial cells [40], of which 7% are microglia and related immune cells [41], the infected CNS could provide a formidable genomic reservoir with more than one million of the intact viral copies.

The study by Plaza–Jennings et al. used a linker-mediated PCR strategy to capture host genome sequences flanking the viral LTR, with the goal to survey on a genome-wide scale the genomic integration sites in postmortem brain. They studied 25 HIV + brain donor subjects with and without encephalitis [23], included 6 uninfected donor brains plus additional positive and negative controls from the cell culture, including lymphocyte clones with single integration sites for technical quality check. The authors collected nuclei by FACS after immunotagging with the neuronal marker NeuN and thus generated integration site maps separately for the neuronal and non-neuronal nuclei population from frontal cortex gray and white matter. Plaza–Jennings et al. captured a total of 1254 integration sites, of which the overwhelming majority, or 97% were derived from non-neuronal brain DNA, primarily from subjects diagnosed with HIV encephalitis [23] together with a more minor contribution from HIV^+^ non-encephalitic donors [23]. The authors analyzed the genomic features of their brain integration sites and compared them to published and newly generated integration site maps from peripheral lymphocytes. Interestingly, the general preference of the virus, to insert itself into a chromatin environment that is “open” as defined by low nucleosomal density and high levels of transcriptional histone marks (including histone lysine acetylation), was apparent in both brain and blood. For example, in both tissues, integration sites were on average located ~ 10^4^ base pairs from the nearest open/facultative chromatin mark, which is two to four orders of magnitude closer than the nearest repressive chromatin mark because those were located ~ 10^6^–10^8^ bp up- or downstream from integration sites [23].

**Fig. 2:**
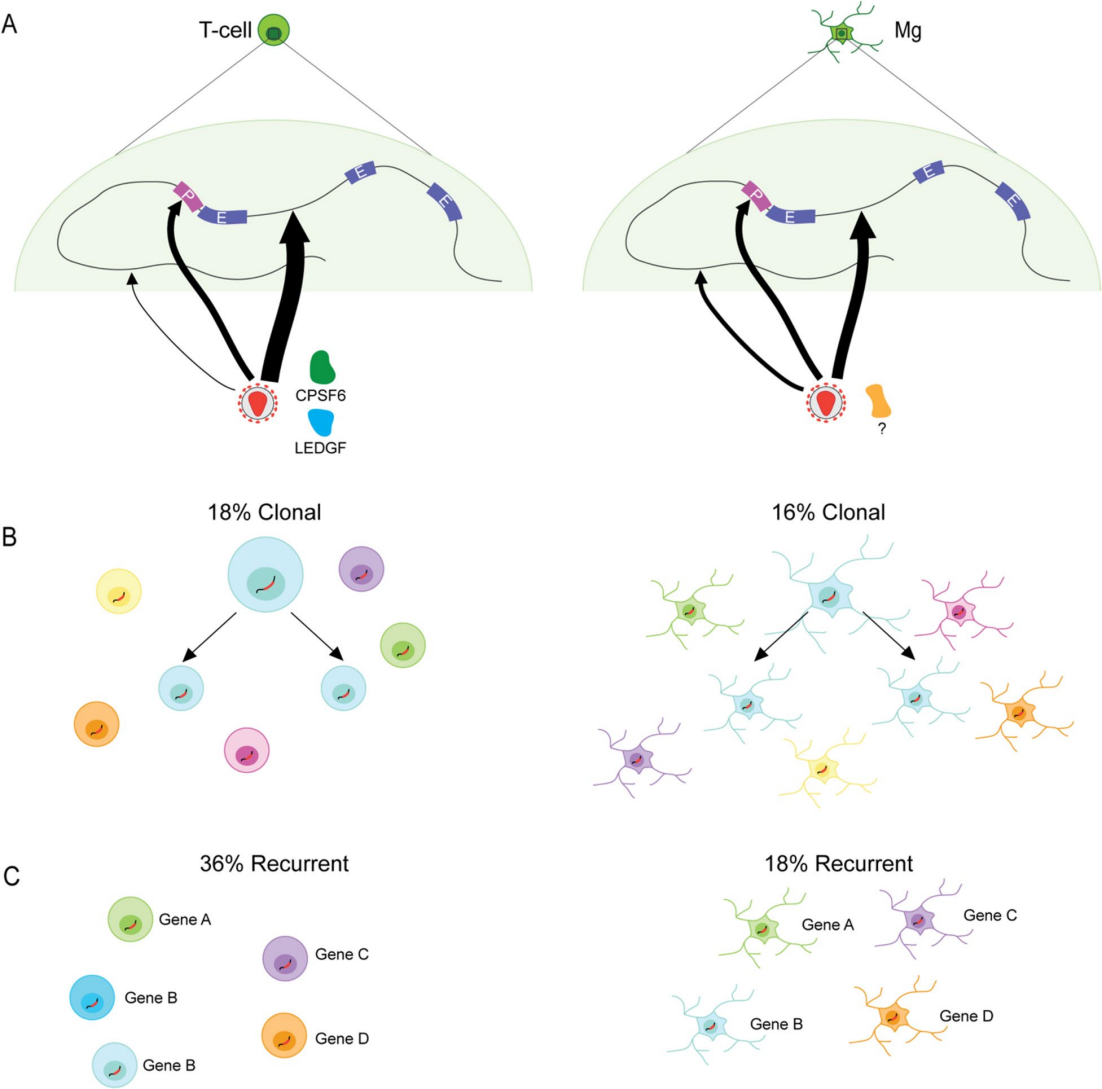
HIV integration site mapping in the brain vs. T-cells. **A** Integration site selection in the brain and in T-cells. In T-cells, CPSF6 and LEDGF guide integration site selection, but this has not yet been shown in microglia. Integration sites are found predominately in intergenic regions, promoter (P) proximal regions, and introns. Very few are found in exons (E). Relative frequencies of integration events into different genomic features are demonstrated by the thickness of the arrows. **B** Clonal integration sites, from the replication of an infected cell, are found at similar frequencies in the brain and in T-cells. **C** Recurrent integration sites, or two independent integration events into the same gene, are more common in T-cells than in the brain

On the other hand, the study reported some notable, genome-scale differences between brain- and blood-specific viral integration (Fig. 2). For example, while both brain and blood showed an over-representation of promoters and gene bodies, reflecting the general predilection of retroviral DNA for sites of transcription, intergenic DNA was targeted at a significantly higher rate in the brain (16.2%) as compared to blood (10.4%). The authors speculate that these higher rates of integration into intergenic regions in brain may be caused by lower levels of expression of the integration targeting factors, *LEDGF* and *CPSF6*. Indeed, in macrophages, lower rates of genic integration have been linked to low levels of expression of *LEDGF *[42], and furthermore, experimentally induced knockout of *LEDGF* in macrophages leads to much higher rates of intergenic integration [2, 3]. These tissue-specific differences are not just for academic reasons of interest, given that differences in integration site targeting could have implications for the development of HIV cure strategies as latency-reversing agents used in shock-and-kill approaches have different efficacy depending on the integration site [43].

### Future Work

While the above-discussed studies on the proviral genome [24, 39] and integration site [23] sequencing from the human brain tissue provide an extremely valuable first glimpse into the interaction between HIV and the brain’s genome, much additional work will be required given the technical limitations of the work conducted thus far. These include insufficient resolution by cell type and failure of the Intact Proviral DNA Assay (IPDA) in the context of viral polymorphisms [44], and overall, low sensitivity (estimated at 1.6%) of linker-mediated PCR assays, which by sequencing out of the viral LTR which also lack information on the sequence of the proviral integrant itself [23]. While the alternative approaches currently available, including the widely used Full-length individual provirus sequencing (FLIPS) assays [36, 37], each has their own advances and limitations, we believe it will be important and worth the considerable expense to map integration sites including their flanking sequences in a highly cell- and tissue-specific manner by long-read DNA sequencing. Such types of techniques are now being imported to the field of retrovirology [45] including HIV research [46], and the field eagerly is awaiting a comprehensive atlas for full-length integration sites.

## Cell-Type Specific Genome-Scale Transcriptome and Epigenome Mapping in the Infected Brain—Emerging Findings

### Current Knowledge

In the general literature, the most frequently mentioned rationale for embarking on transcriptomic and epigenomic studies of the HIV-infected (human) brain refers to a still poorly understood constellation of syndromes, commonly referred to as HIV-associated neurocognitive disorder (HAND), worldwide potentially affecting 20–50% of the 37 million PLWH [11, 12, 47]. This includes the milder forms of HAND in the era of cART. It is important to emphasize that neurocognitive impairment and other manifestations of these “milder” forms still carry the potential to significantly disrupt daily living in 10–12% of PLWH [48]. Therefore, the neurogenomic exploration of postmortem brain from HIV + donors, including those with documented HAND, bears the chance to gain new insights into the underlying molecular and cellular mechanisms. Furthermore, it will be important to study genomic alterations in cases with HIV encephalitis, a condition defined by widespread, substantial neuroinflammation throughout the brain, commonly associated with dementia and severe immunosuppression [11, 12, 47]. Notwithstanding the fact that the prevalence of HIV encephalitis declined after the introduction of combination antiretroviral treatment regimens, it should be viewed as an area of study that continues to be important for HIV postmortem brain research, in order to gain insight into the mechanisms of active retroviral infection and productive CNS invasion in the infected human brain.

Some of the first transcriptomic studies in HIV-infected brain used RNA extracted from bulk tissue as input material. These included cases with encephalitis, and one of the main findings that emerged was that HIV RNA levels correlate with increased expression of pro-inflammatory genes across brain regions [49, 50]. Encephalitis is a frequent long-term complication of HIV infection in the absence of an effective antiretroviral shield, specifically at an advanced stage defined by destruction of the CD4 T-lymphocyte pool and progressively worsening of viremia, the two conditions typically setting the stage for HIV encephalitis [51]. Encephalitic compared to non-encephalitic infected brain tissue displayed elevated expression of interferon (IFN) response genes, genes involved in antigen presentation, complement pathways, and pattern recognition [52]. Specifically, type I (TI) and type II (TII) IFNs and major histocompatibility complex class I (MHC-I) genes were upregulated [53, 54]. Pathways downregulated in HIVE relate to bioenergetics (electron transport chain, tricarboxylic acid cycle) and neuronal functions, while neurodegenerative pathways were generally dysregulated [49, 52, 54]. This overall suggests a shift away from homeostatic functions to inflammation and disruption of neurologic function. However, since these studies were done using RNA extracted from homogenized brain tissue, it is difficult to discern which cell types contribute to these transcriptional alterations in bulk tissue.

To this end, a more recent study gained cell-type-specific resolution of transcription in HIV-infected and uninfected brain by using the 10X Chromium single nucleus RNA-sequencing (snRNA-seq) technique. That study [23] focused on frontal cortex gray matter and underlying subcortical white matter (FC/WM), of 3 uninfected controls, 3 HIV-infected (HIV +) and 7 HIV encephalitis brains. By using stringent quality controls, including wildtype mouse nuclei intermixed with their human samples as additional technical controls to monitor for spurious HIV RNA contamination in the snRNA-seq assay, the authors reported that in the encephalitic brain, 4.8% of all microglial nuclei (including a rare subtype “immune oligodendroglia”) were HIV RNA + , while the proportion of astrocytes, lymphocytes, and neurons harboring HIV RNA-expressing nuclei was lower by at least one order of magnitude (~ 0.1–0.3). Remarkably, a subset of activated microglia in the encephalitic brains expressed dramatically high levels of HIV transcript, surpassing the overall pool of microglia levels of all endogenous transcripts, with the exception of the extremely highly expressed non-coding RNA *NEAT1 *[23].

Consistent with these findings, *CD4* RNA encoding the primary viral receptor [55] was only detectable in the microglial nuclei, and furthermore, expression of *CCR5* and *CXCR4*, the two major viral co-receptors [56], was low across all different cell types, with the exception of robust *CXCR4* expression in the lymphoid cell nuclei [23]. Therefore, these types of transcriptome profiling at single nucleus resolution generally reaffirm that microglia is the primary cell type in the infected brain expressing HIV. However, the low sensitivity of the snRNA-assay as conducted in ref. [23] is noteworthy, given that in contrast to sensitive PCR methods confirming HIV DNA in 3/3 HIV + (non-encephalitic) brains tested, HIV transcript levels in any single nucleus from these infected, non-encephalitic brains from subjects on combination antiretroviral therapy were too low to be detectable. Furthermore, it has been pointed out that nuclear transcriptomics may carry only limited information as it pertains to measuring HIV infection and activity, because intact HIV RNA is post-splicing likely to be rapidly exported from the nucleus to the cytoplasm by the viral protein, Rev [57]. If only Rev-defective viral RNA remains in the nucleus, then single nuclei RNA-seq assays as in ref [23] cannot differentiate between HIV + cells containing intact versus defective virus [57]. To overcome this type of limitation, one would have to study a fresh brain at autopsy, as mentioned above, to isolate intact cells.

The same study [23] also mapped chromosomal conformations on a genome-wide scale specifically in microglia and, separately, neuronal nuclei. The overarching rationale was that combined analysis of cell type-specific gene expression and chromosomal organization could improve functional insight into the genomic adaptations associated with HIV infection in the brain. Chromosomal conformation, or “3D genome” mapping, which by DNA-DNA proximity assay informs about the folding and looping patterns of chromatin, typically at multikilo- to megabase resolution, has been used to computationally dichotomize the nuclear genome into “A” compartments enriched for sites of active gene expression within a loose, permissive chromatin environment. This chromatin state is opposed to the more condensed environment of “B” compartment, primarily linked to less active, or fully silenced chromosomal territories. For recent reviews on the general 3D genome (incl. chromosomal compartment) regulation in the brain, see [58].

Of note, earlier studies had shown that peripheral immune cells undergoing activation tend to display broad changes in chromosomal conformation in the acute cell culture model [59–63], hinting at the possibility that the 3D genome in microglia of the HIV-exposed brain, just like myeloid and lymphoid cells in cell culture, could show adaptations in 3D genome organization as part of the response to viral infection. Indeed, Hi-C chromosome conformation mapping in microglial nuclei from HIV-infected brain uncovered significant changes in A/B compartmentalization [23]. These alterations, while present to a milder extent in HIV^+^ non-encephalitic, were much more profound in HIV^+^ encephalitic brains, and encompassed a total of up to 196 Mb, or 6.4%, of genome sequence (equivalent to the length of human chromosome 5!) as compared to the baseline condition (microglia from uninfected control brain). These changes were highly cell-type specific and not observed in neurons that were FACS-collected from the same tissue block as the sorted microglia. Of note, chromosomal domains that showed increased A compartmentalization in microglia from encephalitic brain included a total of 1940 up-regulated microglial genes, enriched in immune functions such as cytokine signaling, viral response, inflammatory pathways, and T-cell differentiation pathways, chemotaxis, IFN, and interferon and interleukin pathways [23]. Importantly, there is evidence that viral integration shows a preference for such types of genomic loci that become newly activated in the context of neuroinflammation. Thus, it is possible that inflammation and immune activation are not only associated with widespread genomic reorganization in susceptible brain cells but also carry the potential to foster, in a vicious cycle, further spread of infection leading to even more widespread neuroinflammation and encephalitis.

Hi-C 3D genome chromosomal conformation mapping and single nuclei transcriptomic profiling in HIV + non-encephalitic brain provided evidence for much more subtle alterations compared to cases afflicted by more advanced stages of neuroinflammation (encephalitis) [23]. However, microglia from HIV + non-encephalitic brains fully shared with their counterparts from HIV + encephalitic brains a very specific type of genomic alteration. This type of alteration is defined by downregulated expression, and repressive chromosomal (“B”) compartmentalization, for microglial gene sets regulating neuronal and synaptic connectivity [23]. This includes, for example, the Netrin-G1 ligand *LRRC4C/NGL1*, which has been implicated in microglial accumulation at axonic compartments of cortico-spinal projection neurons [64], and genes such as *BCL2L1*, *PNOC*, and *PARKN/PARKIN*, which encode regulators of auto- and mitophagy implicated in synaptic plasticity and neuronal injury and metabolism [65–67]. Based on these preliminary findings then, and pending confirmation in larger scaled studies, the transcriptomic signatures of HIV infection of the brain, including in immunocompetent cases with (near) normal levels of CD4 blood lymphocytes, could be associated with deficits in microglial expression of genes relevant for (microglia-mediated) homeostasis at neuronal synapses. Therefore, a disruption in microglia-dependent neuronal support function may drive, at least in part, the cognitive and neurological deficit in PLWH, including those diagnosed with milder versions of HAND (Fig. 1B, C). However, once the illness advances to more severe stages of neuroinflammation with more rampant viral infection and clinical and histological manifestation of encephalitis, the microglial genome then undergoes a much more massive genomic reprogramming, affecting hundreds of mega-bases of genome encoding immune activator and cytokine signaling-dependent pathways [23]. These changes then are likely to further compromise microglial support functions at the neuronal synapse, with the end of the result of potentially severe dementia [68].

The fact that alterations in microglial transcriptomes and chromosomal conformations in the brain of PLWH otherwise stably maintained on antiretroviral drugs could indicate microglial dysfunction at the level of the neuronal synapse is fully in line with the perceived dual role of microglia as regulators of synaptic transmission, plasticity and pruning, and as modulators of neuroinflammation. These important roles of microglia are perhaps best exemplified by the functional interplay of the neuronally expressed, membrane-bound, and secreted chemokine CX_3_CL1 and its cognate receptor CX_3_CR1 expressed specifically by microglia, and like-wise, by the neuronally expressed classical complement factors such as C1q and C3 that are recognized by the CR3 receptor on microglial membranes [69]. Recent studies, using light sheet fluorescent microscopy and other advanced imaging approaches, have further highlighted the dynamic nature of microglia–synapse interactions, including partial phagocytosis, or trogocytosis (trogo-: nibble), of presynaptic structures and the induction of postsynaptic spine head filopodia by microglia [70]. It is now thought that microglia exert powerful neurophysiological effects on neuronal activity [71], which includes suppressive feedback loops triggered by microglial catabolism of activity-dependent extracellular ATP [72]. Given these findings, it is therefore very conceivable that loss of neuronal support function due to transcriptomic alterations in the microglia from PLWH, including patients taking antiretrovirals, are detrimental to brain function and cognition as manifested in cases diagnosed with the non-dementing “milder” versions of HAND.

### Future Work

Single cell or single nuclei studies of much larger numbers of postmortem brains from donors with HIV are crucially needed to further delineate the cell-type-specific alterations that may occur in the HIV-infected brain. Such large-scale studies are underway, such as the National Institute for Drug Abuse funded Single Cell Opioid Responses in the Context of HIV (SCORCH) project, in which single nucleus RNA-sequencing of a large number of brains is being performed to study the interaction between HIV and drug abuse [73]. Single cell technologies now also allow for the sequencing of epigenetic states (single-cell ATAC-seq) and protein expression (ECCITE-seq). Cell-type specific epigenetic profiles can be generated from nuclei, but studies of protein expression would be best performed on intact cells from fresh postmortem brain. Both approaches will help to deepen our understand of how HIV infection impacts the brain and leads to neurologic dysfunction. For cells of particular interest, more in depth genomic, transcriptomic, and proteomic studies may be performed by pairing these technologies with fluorescence activated cell or nuclei sorting to enrich for cells of interest. Flow-FISH approaches previously discussed to enrich for HIV-infected cells may also be of use to enrich for HIV-infected cells in these analyses.

Another avenue of promising future research is spatial transcriptomic analysis. Beyond cell-type-specific understanding of gene expression, there is another important layer of spatial relationships. The brain is highly structured with cortical layers and different subcortical structures. There is likely to be important information that can be found by looking at cells in their native positions to better understand dysfunction. Especially since data point to a dysfunction in the microglial-neuronal interaction, it would be valuable to study these interactions with spatial relationships maintained. Spatial transcriptomics have been successfully applied to human postmortem brain previously [74]. Thus, this would be possible in the HIV-infected brain and has the potential to yield novel insight.

## Synopsis and Outlook

A thorough genomic exploration of the HIV-infected human brain, in cell (type) specific fashion, including cell specific mappings of transcriptomes and epigenomes, at base-pair resolution on a genome-wide scale, and similarly, of viral integration sites, has barely began. Such type of endeavor should be considered a high priority for government-based and privately organized funding agencies alike. This line of work would be important not just for academic reasons such as gaining more knowledge on the neurobiology of retroviral infection, but also because of pressing public health issues.

This includes our current lack of mechanistic knowledge about neurological and cognitive impairments in the subset of PLWH that are otherwise stably maintained on antiretroviral drug regimens and whose brain-related symptoms are not accompanied by any signs on ongoing peripheral infections. Based on transcriptomic and epigenomic studies on a small number of brains from HIV + donors, compared to uninfected controls, it appears that loss of microglia-mediated homeostatic mechanisms at neuronal synapses is a type of abnormality that is encountered across a wide range of disease stages and that could affect PLWH taking antiretroviral drugs, thereby offering a cellular mechanism underlying HAND even in cases whose systemic infection appears under control.

Furthermore, given the findings from the small number of subjects considered cured from their HIV infection after having received a DCCR5 bone marrow transplant (see above), we would like to be carefully optimistic about the prospect that the brain’s myeloid compartment including microglia by itself, in the absence of an infected peripheral hematopoietic system, cannot initiate reseeding and progressive viral spread. However, much more thorough genomic and functional/virological assessment of the HIV + postmortem brain will be required to test this hypothesis conclusively.

Finally, as discussed in this review, there is increasing evidence that HIV’s genomic integration site selections, and perhaps even some of the host proteins hijacked by the virus’ genomic integration machinery, is different for brain as compared to peripheral blood. It will be important for future study to disentangle in cell-type and tissue-specific fashion the specific subtypes of genomic integration patterns.
